# Herbs Used in Antimalarial Medicines: A Study in the Greater Accra Region of Ghana

**DOI:** 10.1155/2023/6697078

**Published:** 2023-08-19

**Authors:** Nathaniel Nene Djangmah Nortey, Samuel Korsah, Miriam Tagoe, John Antwi Apenteng, Fredrick Akuffo Owusu, Josephine Oppong, Anita Etornam Attah, Sheila Allotey

**Affiliations:** ^1^University of Health and Allied Sciences, Institute of Traditional and Alternative Medicine (ITAM), Ho, Ghana; ^2^Department of Pharmaceutical Sciences, School of Pharmacy, Central University, Accra, Ghana; ^3^Department of Pharmaceutical Sciences, Kwame Nkrumah University of Science and Technology (KNUST), Kumasi, Ghana; ^4^Ho Teaching Hospital, Ho, Ghana

## Abstract

**Methods:**

Pharmacy shops were randomly scouted and products were observed. The active ingredients were documented and their frequencies were determined.

**Results:**

Forty-four (44) plant species belonging to twenty-eight (28) families were recorded for the treatment of malaria in the survey. The predominant families were the Leguminosae and Meliaceae families. *Cryptolepis sanguinolenta (Ghanaian quinine* or yellow dye root) and *Azadirachta indica (*neem tree) were the most cited plants. *Cryptolepis* and neem tree were used 17 and 15 times, respectively, in the finished herbal products for treating malaria. *Conclusion. Cryptolepis sanguinolenta* and *Azadirachta indica (*neem tree) are important herbs for the treatment of malaria in Ghana. Locally manufactured herbal antimalarials are important for the treatment of malaria in urban and rural communities in Ghana.

## 1. Introduction

Malaria is a major health problem worldwide, affecting nearly half of the world's population in 2020, with most cases and deaths occurring in Sub-Saharan Africa and south-eastern Asia [1]. Globally, an estimated 241 million cases of malaria were recorded, with 627,000 of these cases leading to death. The African region shared 95% of the cases recorded worldwide and also 96% of deaths [2]. Ghana is ranked among eleven other countries as a high-burden country, accounting for >70% of the global malaria cases and deaths. In Africa, Ghana is among the two highest-burden countries, reporting the highest absolute increase in malaria cases in 2018 compared to 2017 [3]. However, malaria deaths have significantly reduced in recent times.

The World Health Assembly continues to prioritize the treatment and elimination of malaria in member countries. As part of their efforts to control malaria, in May 2015, the World Health Assembly adopted a global technical strategy for malaria to be implemented between 2016 and 2030. Member countries pledged to accelerate the progress towards malaria elimination by targeting to reduce global malaria incidence and mortality rates by at least 90% by 2030 [4]. As updated in 2021, the group prioritizes ensuring access to malaria prevention, diagnosis, and treatment as part of universal health coverage. Hence, early diagnosis followed by prompt effective treatment of malaria in public and private health facilities and at the community level was adopted as the first pillar. Early diagnosis and treatment of malaria reduce the incidence of the disease and death, further reducing transmission of the parasite [5].

Malaria is solely caused by an infection of the *Plasmodium* parasite through the bite of a female Anopheles mosquito. Currently, five species of *Plasmodium protozoa* (*P. falciparum, P. vivax, P. ovale, P. malariae, and P. knowlesi*) are known to cause malaria worldwide [6]. As recommended by WHO, malaria is treated by artemisinin-based combination therapy (ACT) worldwide and in Ghana [7]. However, there are various home-based antimalarials certified by the Food and Drugs Authority (FDA), widely marketed and used for the treatment of malaria as alternatives or complementing orthodox medications. They are finished herbal products from locally known herbs that have been used in most traditional areas. These products are preferred and highly patronized by Ghanaians, especially by those who perceive herbal medicines to be effective and have less toxic effects. Moreover, although equally effective, some of these products are relatively cheaper and more available in the local communities than orthodox medications. Their compositions are based on oral traditional knowledge of plants in various communities; hence, these herbal products vary widely in their plant compositions.

This study sought to identify plants used in herbal antimalarial preparations in Ghana, determine whether there are scientific data to support their usage in the preparations, and briefly examine if these products meet guidelines certified by the Food and Drugs Authority to be marketed locally and abroad.

## 2. Methodology

### 2.1. Study Design

This study constitutes a survey, categorically nonprobability sampling technique, and convenient sampling, in which readily and accessible community pharmacies in Greater Accra were selected. The major advantages of convenient sampling include its simplicity, cost-effectiveness, and less time consumption [8]. A total of ninety (90) pharmaceutical outlets where finished herbal medicinal products are sold were visited with the aim of collecting data on the products being sold and observing the label for constituents (plants used) to determine the most frequently used plants in local antimalarial herbal preparations being marketed. The individual plant species were grouped into their respective plant families and their frequency of use was recorded. In addition, the labels were observed for the following: manufacturer, date of manufacturing and expiry, batch number, and Food and Drugs Authority (FDA) registration reference as required. The names of the pharmacies where the products were sampled as well as their global positioning system coordinates (GPS) using the GPS application were documented. The data accumulated were analyzed using Microsoft Office (Excel) 2007.

### 2.2. Ethical Considerations

In carrying out this study, permission was sought from management or pharmacists or attendants of pharmacies that had finished herbal products on their shelves for consumers. The concept and purpose of the research were explained to them to receive clearance.

### 2.3. Study Population

The study was conducted in 10 districts in Greater Accra, namely, Ashaiman Municipal District, Tema Metropolitan District, Ningo-Prampram District, Ledzokuku-Krowor Municipal District, Labone Constituency, Accra Metropolitan District, Tema West Municipal District, and Adenta Municipal District, from February to March 2022. The targets were community pharmacies that sold herbal antimalaria products used for the treatment and management of malaria. These community pharmacies were known to be the source of primary healthcare in their respective districts.

Greater Accra is the smallest of Ghana's sixteen administrative regions. It has a total land surface area of about 3,245 square kilometres, representing 1.4% of the country's total land area. Despite its size, it is the second-most populated region in the country after the Ashanti Region [9]. According to the 2021 population and housing census, the Greater Accra Region has a population of 5,455,692 Ghanaians from all over the country representing 17.7% of the country's population [10]. Tema is a city on the Ghanaian coastal plain. It is 25 kilometres east of Accra and had a population of 161,612 in 2013 [11]. It is the second largest city in the Greater Accra Region after Accra. It is also the industrial hub of Ghana as well as home to the country's largest port in Tema Harbour. It is also known as the hotspot of commercial activities. These two cities were chosen for the study because they are home to many people of different ethnic backgrounds and are hotspots for commercial activities.

## 3. Eligibility Criteria

All wholesale and retail pharmacies as well as licensed chemical shops in the Greater Accra Region qualify to be part of this study. In Ghana, individuals who are not pharmacists but have been certified by the Pharmacy Council (a regulating body on medicines) can sell Class C (over-the-counter) drugs in what is commonly termed as Licensed Chemical Shops to the general public, especially in rural areas.

## 4. Data Collection

The outlets were visited and the information was gathered by direct observation of antimalarial herbal products with the assistance of an attendant. The information was directly entered into Google forms already designed.

### 4.1. Data Analysis

The data collected were analyzed into tables and graphs using Microsoft Excel, 2016. The names of the outlets visited and their GPS were recorded. The GPS was converted to coordinate and represented on a map using ArcGis 7.0. Finished antimalarial herbal products were also countered and the respective constituents were documented by recording their botanical names as it appeared on the label.

## 5. Results and Discussion

Previous studies have documented over 1,200 plant species from 160 families used in the treatment of malaria or fever. In Ghana, some ethnobotanical studies have been conducted on herbal therapies used for the treatment of malaria in different communities and localities [12]. These studies have mostly focused on plants purported to have antimalarial activities; few, however, have focused on the herbal constituents of commercial antimalarial herbal mixtures.

**Figure 1:**
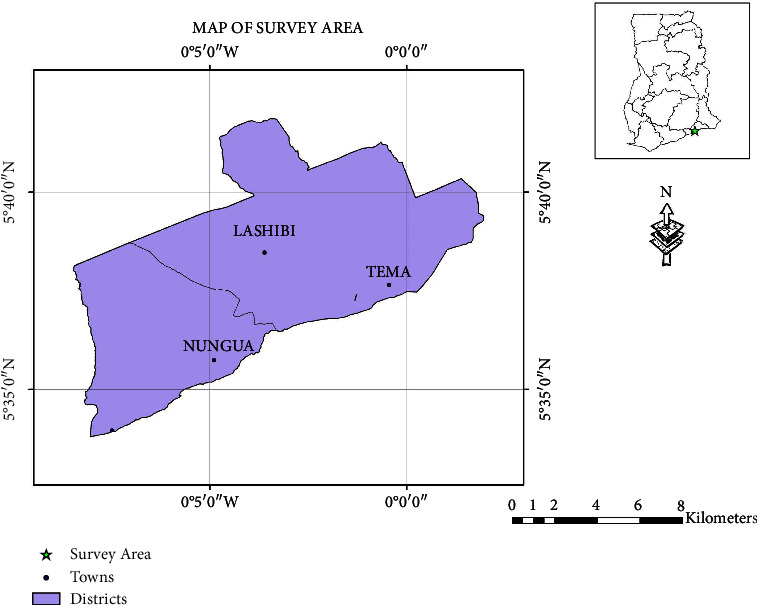
Map of Ghana showing the selected area of the survey in GPS coordinates.

In all, a total of 90 pharmacies and licensed chemical outlets were randomly scouted within the Greater Accra Region. The GPS coordinates of the pharmacies were extracted and presented graphically (Figure 1). The labels indicated that all products were locally manufactured by industries based in Ghana. A total of 53 different antimalarial herbal products were sampled from 90 outlets (Table 1). The active ingredients of the various products as inscribed on the label were listed as seen in Table 1.

**Figure 2:**
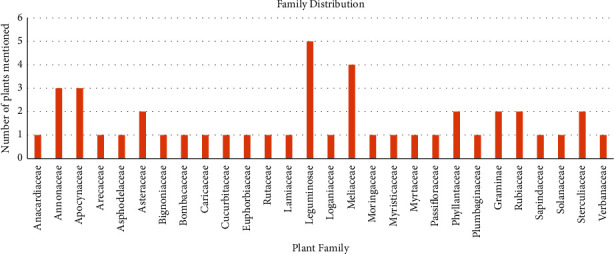
Frequency distribution of families.

Plant names (botanical names) were documented and their frequency in various products was recorded as well (Table 2). Only six (6) products were formulated from one plant, while forty-seven (47) products had two or more active ingredients (Table 2). The various products had varying active constituents between 1 and 6 per information on the label. Forty-six (44) different plants were identified in the products belonging to twenty-eight (28) families. *Cryptolepis sanguinolenta* (Lindl.) *Schlechter* (Apocynaceae) and *Azadirachta indica* A. (Juss) were documented 17 and 15 times, respectively, emerging as the most used plants by producers. These were followed by *Alstonia boonei* (12), *Morinda lucida* (6), *Citrus aurantifolia* (7), *Paullinia pinnata* (7), *Tetrapleura teptratera* (7), and *Vernonia amygdalina* (6) as illustrated in Figure 2.

*C. sanguinolenta* has countless reports on its antimalarial properties. The plant is locally referred to as Nibima (Twi) and is commonly known as the West African antimalarial herb [13]. Lepiquin, Nolico mixture, Masada mixture, and Nibima herbal medicine were produced solely from this herb. Despite its reported antibacterial, anticancer, and antiinflammatory properties, the plant is most widely regarded for its antimalarial properties [14]. The antimalarial activity of the plant has been linked to its main alkaloid cryptolepine and its analogues (cryptoquindoline, quindoline, biscryptolepine, and cryptospirolepine) isolated from the plant roots [15]. A decoction of the root showed comparable efficacy to chloroquine in a small open randomized clinical trial in adults [16]. More importantly, its major alkaloid, cryptolepine, has shown potent *in vitro* antiplasmodial activity against chloroquine-resistant strains of *P. falciparum* [17]. Hence, the folkloric use of *C. sanguinolenta* has much scientific evidence to support its use in the treatment of malaria. Although the compound responsible for the activity is known, there is no locally manufactured product from the compound for the treatment of malaria. A decoction of the roots is preferred.

*Azadirachta indica* A. (Juss), commonly known as the neem tree (Kingtso-Ga, Liliti-Ewe, and Dua gyanne), is known for its multifaceted healthful properties. The leaf, seed, and stem bark extracts of *A. indica* have shown *in vitro* inhibitory activity against *Plasmodium falciparum*. Further research on limonoids (gedunin and nimbolide) from *A. indica* has also shown activity against *P. falciparum* [18]. The plant is widely used in Asia (India), Sub-Saharan Africa, and America for the treatment of various diseases and has been nick-named “village pharmacy” (in India) [19]. In India, the fresh leaves are cooked and eaten to gain immunity from malaria [20], whereas in Ghana, steam inhalation of the leaves is used to relieve patients of malaria and its related symptoms. As demonstrated in the study, the decoction of the plant in combination with other herbs is a preferred dosage form. In a study conducted by Braga and associates, neem extracts/subproducts are nontoxic or less toxic when orally administered. Toxicity in animals was, however, reported when administered intraperitoneal [21].

The Leguminosae family recorded 5 active ingredients representing the highest number of plants belonging to a single family. *Pilostigma thonningi*, *Cassia siamea*, *Cassia alata*, *Tetrapleura tetraptera,* and *Albizia ferruginea* were plants of the Leguminosae family listed. Leguminosae, belonging to the Fabales order, is the third largest plant family with over 19,327 plant species and 727 genera. Six subfamilies (Caesalpinoideae, Dialioideae, Dearioideae, Cercidoideae, Duparquetioidae, and Papilionoidae) make up this family [22]. The Meliaceae family includes 4 herbs: *Carapa procera, Khaya senegalensis, Khaya ivorensis,* and *Azadirachta indica*, respectively. Several plant secondary metabolites have been isolated from plants in this family, but significantly triterpenoids and limonoids, which are mostly responsible for the various biological activities [23]. Limonoids are known to have a bitter taste and are responsible for the taste of citrus and plants in this family [24]. Hence, the bitter taste of the neem and *Khaya* species can be attributed to this class of compounds.

A brief review of the constituents listed showed that the majority of them have been scientifically investigated for their antimalarial activity and its related symptoms. The following, however, had no scientific data to support their folkloric use; *Ananas sativus*, *Citrus aurantifolia*, *Vitex grandifolia*, *Albizia ferruginea*, *Cola gigantea*, *Theobroma cacao*, *Solanum torvum*, *Phyllanthus nuriri*, *Aloe schweinfurthii*, *Paullinia pinnata*, *Pycanthus angolesis*, *Adenia cissampeloides*, *Plumbago capensis*, *Cymbopogon citratus*, *Raphia hookeri*, and *Momordica charantia.* However, in herbal medicine, multiple herbs are used in a single preparation with a focus on delivering multifaceted healing to the body. Hence, some plants listed may not have a direct link to malaria but when added will improve the general well-being of patients. This theory is known and widely practiced by herbalists. Moreover, it has been scientifically proven that the actions of some active ingredients are likely to be potentiated or enhanced when used with other herbs. The negative outcome and possible toxicities cannot be ruled out. Arguably, the mere combination of these herbs does not guarantee their optimal activity in therapy. More research into their effective combinations (specific ratios) must be studied to improve their safety effectiveness. These studies will also indicate if the plethora of herbs used in the formulations is necessary or if they could be possibly reduced to achieve optimal results [25].

Moreover, although the active ingredients have been stated, the parts of these herbs used in the formulation are not indicated. Plant parts used must be indicated to give a clear guide to scientists for various studies on the herbs and products. Herbalists are in a habit of concealing this information in the quest to protect their brands from being pirated. However, this information is crucial in the scientific verification of plant use.

It is laudable to note that all but one of the products surveyed have been registered with the Food and Drug Authority based on the registration numbers on the label. However, the FDA does not have a list of registered herbal products on its website for verification. In compliance with good manufacturing practice, all products had clearly stated date of manufacturing and expiry in addition to batch numbers.

It is commendable to note that these formulations have been integrated into the National Health Insurance. Hence, patients who prefer to have herbal remedies can access them through their National Health Insurance. This provides an improved and a cheaper option of obtaining the products for the cure of malaria.

The constant use of these plant materials (*Cryptolepis sanguinolenta* and *Azadirachta indica*) raises concerns about their sustainable use. In the case of *Cryptolepis,* the roots are mostly used which infers the plant is always lost after harvesting and must be replaced [26]. Constantly harvesting them from the wild can endanger the species and ultimately make them extinct. Article 7 of the Convention on Biological Diversity seeks to identify components of biological diversity important for its conservation and sustainable use. Article 8(a) also seeks to establish a system of protected areas or areas where special measures need to be taken to conserve biological diversity. The use of these plants (*Cryptolepis* and neem tree) requires that they are protected and conversed for sustained use [27]. The labels do not provide information on the source of these herbs for the various preparations. However, they are public institutions, for example, the Centre for Plant Medicine Research which cultivates medicinal plants to feed their drug production unit and also for commercial purposes. Some local farmers also cultivate herbs of commercial value in Ghana. In order to preserve these plants, we recommend that laws should be formulated to preserve forest areas where these plants can be found. These policies should also provide measures to ensure that the plants are replaced even after harvesting.

## 6. Conclusion

Our study on the selected finished antimalarial herbal products in Accra has shown that *Cryptolepis sanguinolenta* and *Azadiracta indica* are the most used plants in these commercial antimalarial herbal products. Several scientific reports also support the use of these plants in the treatment of malaria. The use of *Cryptolepis* over the years proves that it is highly effective in the treatment of malaria. Malaria can be locally managed with these products.

## Figures and Tables

**Table 1:** Some herbal antimalarial drugs with their corresponding active ingredients.

Product names	Plant constituents
(1) Givers herbal mixture	*Khaya senegalensis, Azadirachta indica*
(2) Taabea herbal mixture	*Ocimum viride, Azadirachta indica, Tetrapleura tetraptera, Theobroma cacao,*
	*Cymbopogon citratus, Moringa oleifera*
(3) Tinattet malakare	*Carapa procera, Cryptolepis sanguinolenta*
(4) Angel herbal mixture	*Cola gigantea, Spathodea campanulata, Vernonia amygdalina, Solanum torvum, Bombax buonopozense*
(5) Adutwumwaa malamix	*Phyllanthus fraternus, Anthocleista nobilis, Vitex grandifolia*
(6) Top fever mixture	*Alstonia boonei, Azadirachta indica*
(7) Masada mixture	*Cryptolepis sanguinolenta*
(8) Time herbal mixture	*Cola gigantea, Spathodea campanulata, Bombax buonopozense, Vernonia amygdalina*
(9) Rooter mixture	*Aloe schweinfurthii, Khaya senegalensis, Pilostigma thonningii, Cassia siamea*
(10) Amuzu herbal mixture	*Spathodea campanulata, Alchornea cordifolia, Paullinia pinnata*
(11) Lawson herbal mixture	*Khaya senegalensis; Rauwolfia vomitoria; Alstonia boonei; Pycnanthus angolensis*
(12) Agbeve tonic	*Xylopia aethiopica, Citrus aurantifolia, Psidium guajava, Bidens pilosa, Paullinia pinnata*
(13) Osompa *t*-malamix	*Cassia alata, Carica papaya*
(14) Yaakson mixture	*Paullinia pinnata, Khaya ivorensis, Mangifera indica*
(15) Ibrahim herbal mixture	*Rauwolfia vomitoria, Albizia ferruginea*
(16) Adom mala mixture	*Cryptolepis sanguinolent, Azadirachta indica*
(17) Brs mala mixture	*Morinda lucida, Acacia siamea, Vitex grandifolia*
(18) Ebenezer favare mixture	*Alstonia boonei, Morinda lucida, Rauwolfia vomitoria, Tetrapleura tetraptera*
(19) Ebetoda bitters	*Cassia siamea, Pilostigma thonningi, Azadirachta indica, Khaya senegalensis*
(20) Eff's malamix	*Nauclea latifolia, Cryptolepis sanguinolenta*
(21) Fosuaa herbal mixture	*Cassia alata, Morinda lucida, Tectona grandis, Carica papaya, Citrus aurantifolia*
(22) Aduana mala 4	*Alstonia boonei, Acacia siamea, Ocimum gratissimum*
(23) Sibi malacure	*Citrus aurantifolia, Azadirachta indica*
(24) Nibima herbal medicine	*Cryptolepis sanguinolenta*
(25) Herbaquin	*Xylopia aethiopica, Cryptolepis sanguinolenta, Alstonia boonei, Monodora myristica, Azadirachta indica*
(26) Maame dagomba da hiada herbal mixture	*Moringa oleifera, Paullinia pinnata, Cassia alata, Azadirachta indica, Tetrapleura tetraptera*
(27) Adusa malaherb mixture	*Vernonia amygdalina, Azadirachta indica, Morinda lucida, Morinda oleifera*
(28) Okonkwo herbal mixture	*Xylopia aethiopica, Paullinia pinnata*
(29) Dannemal herbal mixture	*Rauwolfia vomitoria, Pycnanthus angolensi, Paullinia pinnata, Khaya ivorensis, Mangifera indica*
(30) Lucky herbal mixture	*Moringa oleifera, Enantia polycarpa, Adenia cissampeloides, Plumbago capensis, Tetrapleura tetraptera*
(31) Aseda herbal mixture	*Alstonia booneI, Mangifera indica*
(32) Gifas malaria mixture	*Vernonia amygdalina, Alstonia boonei, Ocimum gratissimum*
(33) Yafo fever mixture	*Cryptolepis sanguinolenta, Azadirachta indica*
(34) Malaherb	*Cryptolepis sanguinolenta, Azadirachta indica*
(35) Malarigo mixture	*Cassia siamea, Citrus aurantifolia*
(36) Franko herbal mixture	*Carica papaya, Alstonia boonei, Tetrapleura tetraptera*
(37) Plasmox herbal mixture	*Cryptolepis sanguinolenta, Azadirachta indica*
(38) Mala thyphs	*Khaya senegalensis, Cassia siamea, Citrus aurantifolia, Cryptolepis sanguinolenta*
(39) Fosuaa herbal mixture	*Carica papaya, Alstonia boonei, Tetrapleura tetraptera*
(40) Brightfod strong	*Psidium guajava, Citrus aurantifolia, Paullinia pinnata*
(41) Trust herbal mixture	*Pycanthus angolensis, Moringa oleifera, Tetrapleura tetraptera*
(42) Naana herbal mixture	*Alstonia boonei, Ocimum gratissimum, Azadirachta indica*
(43) Danpo herbal mixture	*Spathodea campanulata, Cryptolepis sanguinolenta, Morinda lucida, Alchornea cordifolia*
(44) Gidimal herbal	*Cryptolepis sanguinolenta, Morinda lucida*
(45) Asinku herbal mixture	*Vernonia amygdalina, Raphia hookeri, Alchornea cordifolia*
(46) Lepiquin	*Cryptolepis sanguinolenta*
(47) Nolico mixture	*Cryptolepis sanguinolenta*
(48) Malaquin herbal mixture	*Vernonia amygdalina; Cryptolepis sanguinolenta*
(49) Top tonic	*Alstonia boonei; Khaya ivorensis*
(50) Daprof malamoid	*Citrus aurantifolia, Cryptolepis sanguinolenta, Azadirachta indica, Mangifera indica*
(51) Ab's herbal mixture	*Rauwolfia vomitoria, Momordica charantia*
(52) Golden herbal mixture	*Azadirachta indica*

**Table 2:** Distribution of constituents and their families.

Families	Active ingredients	Frequency of occurrence in preparation
(1) Anacardiaceae	*Mangifera indica*	4
(2) Annonaceae	*Monodora myristica*	1
	Xylopia aethiopica	2
	*Enantia polycarpa*	1
(3) Apocynaceae	*Alstonia boonei*	12
	*Cryptolepis sanguinolenta*	17
	*Rauwolfia vomitoria*	1
(4) Arecaceae	*Raphia hookeri*	1
(5) Asphodelaceae	*Aloe schweinfurthii*	3
(6) Asteraceae	*Vernonia amygdalina*	6
	*Bidens pilosa*	1
(7) Bignoniaceae	*Spathodea campanulata*	4
(8) Bombacaceae	*Bombax buonopozense*	2
(9) Caricaceae	*Carica papaya*	4
(10) Cucurbitaceae	*Momordica charantia*	1
(11) Euphorbiaceae	*Alchornea cordifolia*	3
(12) Rutaceae	*Citrus aurantifolia*	7
(13) Lamiaceae	*Tectona grandis*	1
(14) Leguminosae	*Pilostigma thonningi*	2
	*Cassia siamea*	4
	*Cassia alata*	3
	*Tetrapleura tetraptera*	7
	*Albizia ferruginea*	1
(15) Loganiaceae	*Anthocleista nobilis*	1
(16) Meliaceae	*Carapa procera*	1
	*Khaya senegalensis*	5
	*Khaya ivorensis*	3
	*Azadirachta indica*	15
(17) Moringaceae	*Moringa oleifera*	4
(18) Myristicaceae	*Pycanthus angolensis*	3
(19) Myrtaceae	*Psidium guajava*	2
(20) Passifloraceae	*Adenia cissampeloides*	1
(21) Phyllantaceae	*Phyllanthus fraternus*	1
	*Phyllanthus niruri*	1
(22) Plumbaginaceae	*Plumbago capensis*	1
(23) Graminae	*Cymbopogon citratus*	1
	*Ocimum gratissimum*	4
(24) Rubiaceae	*Morinda lucida*	6
	*Nauclea latifolia*	1
(25) Sapindaceae	*Paullinia pinnata*	7
(26) Solanaceae	*Solanum torvum*	1
(27) Sterculiaceae	*Cola gigantea*	2
	*Theobroma cacao*	1
(28) Verbanaceae	*Vitex grandifolia*	2

## Data Availability

The data used to support the findings of this study are included in the article and available from the corresponding author upon request.
